# Anti-Inflammatory Effects of Tegoprazan in Lipopolysaccharide-Stimulated Bone-Marrow-Derived Macrophages

**DOI:** 10.3390/ijms241914589

**Published:** 2023-09-26

**Authors:** Gong-Ho Han, Seong-Jun Kim, Wan-Kyu Ko, Je-Beom Hong, Seung-Hun Sheen, Min-Jai Cho, Seil Sohn

**Affiliations:** 1 Department of Neurosurgery, CHA Bundang Medical Center, CHA University, 59 Yatap-ro, Bundang-gu, Seongnam-si 13496, Gyeonggi-do, Republic of Korea; hgh429@chauniv.ac.kr (G.-H.H.); ksj987456@chauniv.ac.kr (S.-J.K.); wankyu@chauniv.ac.kr (W.-K.K.); nssheen@cha.ac.kr (S.-H.S.); 2 Department of Life Science, CHA University, Boondagger, Seongnam-si 13493, Gyeonggi-do, Republic of Korea; 3 Department of Neurosurgery, Kangbuk Samsung Hospital, Sungkyunkwan University School of Medicine, Seoul 16419, Republic of Korea; jebeomhong@gmail.com; 4 Department of Neurosurgery, Chungbuk National University College of Medicine, Chungbuk National University Hospital, Seowon-gu, Cheongju-si 28644, Chungcheong-do, Republic of Korea

**Keywords:** tegoprazan, inflammation, anti-inflammation substance, LPS-induced inflammation, bone-marrow-derived macrophages

## Abstract

The purpose of this study was to investigate the anti-inflammatory effect of tegoprazan (TEGO) in lipopolysaccharide (LPS)-stimulated bone-marrow-derived macrophages (BMMs). To this end, compared to methylprednisolone (MP; positive control), we evaluated whether TEGO effectively differentiates LPS-stimulated BMMs into M2-phenotype macrophages. Moreover, the expression of pro- and anti-inflammatory cytokines genes influenced by TEGO was measured using quantitative real-time polymerase chain reaction (qRT-PCR) analysis. TEGO was found to reduce nitric oxide (NO) production in BMMs significantly. In addition, TEGO significantly decreased and increased the gene expression levels of pro-inflammatory and anti-inflammatory cytokines, respectively. In addition, we evaluated the phosphorylated values of the extracellular signal-regulatory kinase (ERK) and p38 in the mitogen-activated protein (MAP) kinase signaling pathway through Western blotting. TEGO significantly reduced the phosphorylated values of the ERK and p38. In other words, TEGO suppressed the various pro-inflammatory responses in LPS-induced BMMs. These results show that TEGO has the potential to be used as an anti-inflammatory agent.

## 1. Introduction

The secretion of inflammatory cytokines is a biological response to external stimuli [1]. This response is a natural process involving immune cells and inflammatory mediators [2]. An abnormal increase in pro-inflammatory factors, including nitric oxide (NO) [3], tumor necrosis factor-alpha (TNF-α), interleukin-1 beta (IL-1β), and IL-6, is characteristic of the inflammatory responses of macrophages [4]. Macrophages exhibit two distinct phenotypes, including M1 and M2 phenotypes [5]. M1-phenotype macrophages produce pro-inflammatory cytokines, exacerbating inflammation [6]. Conversely, M2-phenotype macrophages produce anti-inflammatory cytokines and promote tissue remodeling and repair [7]. These inflammatory factors are induced by the mitogen-activated protein kinase (MAPK) signal pathways, specifically extracellular signal-regulated kinase (ERK) and p38 [8]. The MAPK pathway initiates the downstream induction of nuclear factor kappa B (NF-κB) [9]. NF-κB plays an essential role in inflammation by regulating the expression of numerous cytokines, transcription factors, and regulatory proteins [10].

The excessive secretion of inflammatory mediators is toxic to humans [2]. Although steroidal medicines are used to decrease pro-inflammatory responses, steroids have several side effects, including reduced immunity, which can lead to severe infections [11].

Methylprednisolone (MP) has been utilized in the treatment of patients with inflammatory diseases such as rheumatoid arthritis, inflammatory bowel disease, dermatomyositis, and acute spinal cord injuries [12]. However, long-term/high-dose treatment with MP is associated with side effects including infections, pneumonia, and myopathy [13]. Consequently, non-steroidal anti-inflammatory drugs (NSAIDs) are required to overcome the side effects of the steroid treatments in clinics [14]. 

Tegoprazan (TEGO) is a potassium-competitive acid blocker (P-CAB) used to treat acid-related gastroenteritis [15,16] and gastro-esophageal reflux disease (GERD) [17,18,19]. It has been approved as a treatment for GERD, Helicobacter pylori infections, and gastric ulcers in South Korea [20]. It was also approved for erosive esophagitis in China [20]. Although there are several reports that long-term use potentially increases the risk of hip fractures, it is reported that the potassium channel on macrophages is involved in the activation and the polarization [21]. Based on previous studies, while TEGO has the potential to be used as an NSAID due to its role as a potassium-competitive inhibitor, to the best of our knowledge, there have been no investigations of the effects of TEGO on macrophages.

Hence, we aimed to investigate the anti-inflammatory effects of TEGO in lipopolysaccharide (LPS)-stimulated bone-marrow-derived macrophages (BMMs).

## 2. Results

### 2.1. Effects of TEGO on Cytotoxicity and Nitric Oxide (NO) Production in BMMs

**Figure 1:**
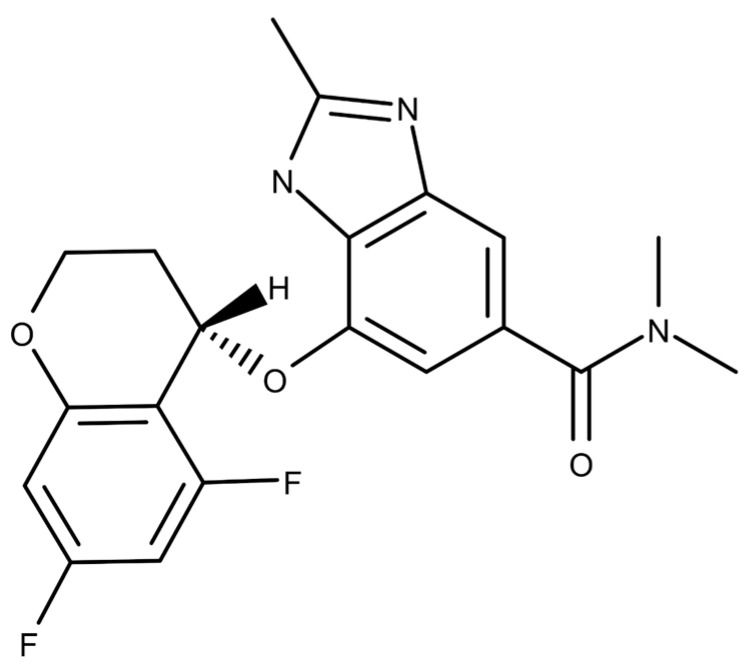
Structure of tegoprazan (TEGO).

**Figure 2:**
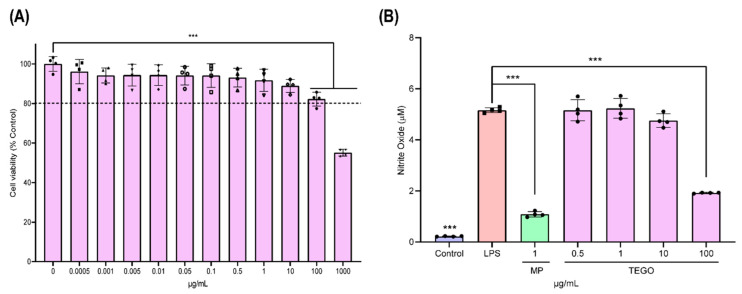
(**A**) Effect of TEGO on the cell viability of bone-marrow-derived macrophages (BMMs). (**B**) Effect on nitric oxide (NO) production in BMMs treated with lipopolysaccharide (LPS), 1 μg/mL methylprednisolone (MP), and TEGO. The results are expressed as the mean ± standard deviation (SD, n = 4 per group); *** *p* < 0.001, a significant difference as compared to the control group and to each group.

Figure 1 presents the molecular structure of TEGO. Compared to the cells of the 0 μg/mL group, cell viability of more than 80% was found at TEGO concentrations of 0.5, 1, 10, and 100 μg/mL in BMMs (Figure 2A, control (0 μg/mL): 100 ± 3.70; 0.5 μg/mL: 93.08 ± 4.70; 1 μg/mL: 91.76 ± 5.56; 10 μg/mL: 88.93 ± 3.28; and 100 μg/mL: 82.25 ± 3.47). However, the cell viability was less than 80% at a TEGO concentration of 1000 μg/mL in BMMs (1000 μg/mL of TEGO: 55.16 ± 1.71, *** *p* < 0.001).

A significant increase in NO production was observed in the LPS group (5.16 μM ± 0.1) compared to that in the control (0.22 μM ± 0.01) (Figure 2B). The NO production values were 5.16 μM ± 0.41, 5.23 μM ± 0.39, 4.75 μM ± 0.26, and 1.92 μM ± 0.01 in the 0.5, 1, 10, and 100 μg/mL TEGO groups, respectively. In the 100 μg/mL TEGO and 1 μg/mL MP (1.08 μM ± 0.11) groups, it was significantly decreased, compared to the LPS group. In other words, NO production was decreased most significantly in the 100 μg/mL group of TEGO.

### 2.2. TEGO Treatment Increases Differentiation into M2-Phenotype Macrophages

**Figure 3:**
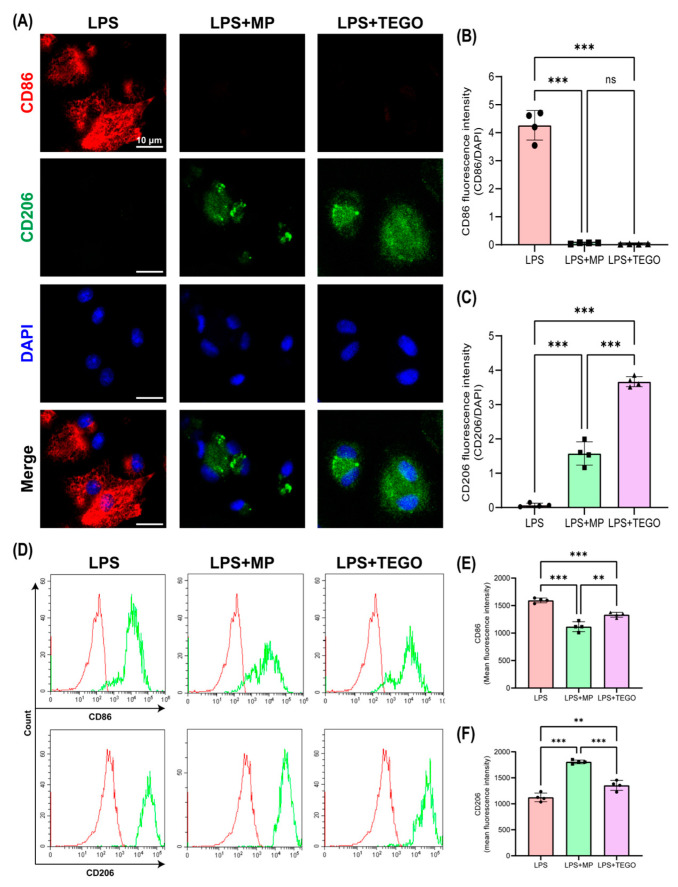
Immunocytochemistry (ICC) staining and fluorescence-activated cell sorting (FACS) of BMMs treated by TEGO. (**A**) ICC staining of cluster of differentiation (CD) 86 and CD206 in groups treated with LPS, LPS+MP, and LPS+TEGO (scale bar = 10 μm). (**B**) Quantitative analysis of the CD86 fluorescence intensity levels. (**C**) Quantitative analysis of the CD206 fluorescence intensity levels. (**D**) CD86-positive and CD206-positive (green) BMM populations selected for analysis among CD68-positive (red). Quantitative analysis of the (**E**) CD86 and (**F**) CD206 fluorescence intensity levels using FACS. The results are expressed as the mean ± SD (n = 4 per group); not significant (ns), ** *p* < 0.01 and *** *p* < 0.001, a significant difference as compared to the control group and to each group.

To investigate whether TEGO upregulates M2 differentiation, we performed immunocytochemistry (ICC) staining on BMMs treated with LPS, LPS+MP, and LPS+TEGO (Figure 3A–C). M1- and M2-phenotype macrophages are commonly associated with the expression of surface antigens, such as cluster of differentiation (CD) 86 (*** *p* < 0.001). Furthermore, the CD206 expression levels in the LPS+MP and LPS+TEGO groups were significantly higher than those for LPS alone (Figure 3C, LPS: 0.07 ± 0.06; LPS+MP: 1.58 ± 0.34; and LPS+TEGO: 3.67 ± 0.15; ** *p* < 0.01 and *** *p* < 0.001).

To determine whether TEGO can induce M2-phenotype macrophages, we conducted fluorescence-activated cell sorting (FACS) analysis (Figure 3D–F). CD68 was utilized as a pan-macrophage marker. The expression levels of CD86 in the LPS+MP and LPS+TEGO groups were significantly lower than those in the LPS group (Figure 3E, LPS: 1594.25 ± 43.28; LPS+MP: 1116.00 ± 89.41; and LPS+TEGO: 1332.75 ± 45.38; *** *p* < 0.001). Conversely, the expression levels of CD206 in the LPS+MP and LPS+TEGO groups were significantly higher than those in the LPS group (Figure 3F, LPS: 1124.00 ± 83.12; LPS+MP: 1807.00 ± 35.58; and LPS+TEGO: 1354.75 ± 95.61; * *p* < 0.01 and *** *p* < 0.001).

### 2.3. Effects of TEGO on mRNA Expression Levels of Pro-Inflammatory and Anti-Inflammatory Cytokine

**Figure 4:**
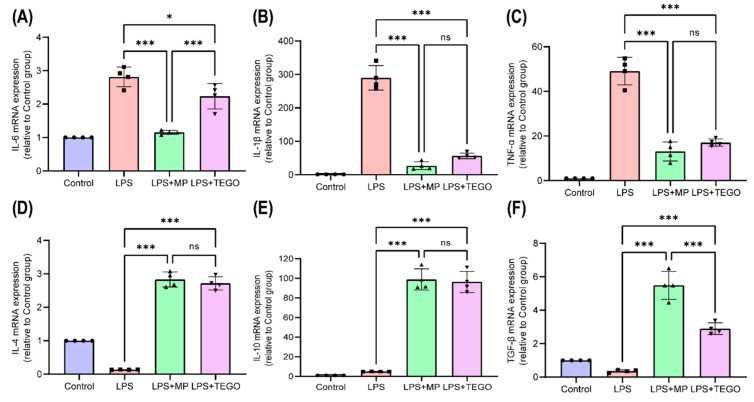
The mRNA expression of pro-inflammatory cytokines (**A**) IL-6, (**B**) IL-1β, and (**C**) TNF-α using TEGO on LPS-stimulated BMMs. The mRNA expression of anti-inflammatory cytokines (**D**) IL-4, (**E**) IL-10, and (**F**) transforming growth factor-beta (TGF-β) using TEGO on LPS-stimulated BMMs. The results are expressed as the mean ± SD (n = 4 per group); not significant (ns); * *p* < 0.05 and *** *p* < 0.001, a significant difference as compared to each group.

The mRNA expression levels of pro-inflammatory cytokines IL-6, IL-1β, and TNF-α are shown in Figure 4A–C, respectively. The average values of IL-6, IL-1β, and TNF-α were highest in the LPS group (Figure 4A–C, IL-6: 2.81 ± 0.26, IL-1β: 289.79 ± 31.68, and TNF-α: 47.18 ± 5.42). The gene expression levels of IL-6 were significantly decreased in the LPS+MP and LPS+TEGO groups (Figure 4A, LPS+MP: 1.16 ± 0.05 and LPS+TEGO: 2.24 ± 0.33) compared to those in the LPS group (2.81 ± 0.26, * *p* < 0.05 and *** *p* < 0.001). Moreover, the gene expression levels of IL-1β were significantly decreased in the LPS+MP and LPS+TEGO groups (Figure 4B, LPS+MP: 26.99 ± 10.07 and LPS+TEGO: 56.46 ± 7.45) compared to those in the LPS group (289.79 ± 31.67, *** *p* < 0.001). The gene expression levels of TNF-α also were significantly decreased in the LPS+MP and LPS+TEGO groups (Figure 4C, LPS+MP: 17.77 ± 3.76 and LPS+TEGO: 17.60 ± 1.43) compared to those in the LPS group (47.18 ± 5.42, *** *p* < 0.001).

The mRNA expression levels of anti-inflammatory cytokines IL-4, IL-10, and transforming growth factor (TGF)-β are shown in Figure 4D–F, respectively. The gene expression levels of IL-4 were significantly increased in the LPS+MP and LPS+TEGO groups (Figure 4D, LPS+MP: 2.83 ± 0.19 and LPS+TEGO: 2.72 ± 0.17) compared to those in the LPS group (0.14 ± 0.01, *** *p* < 0.001). Moreover, the gene expression levels of IL-10 were significantly increased in the LPS+MP and LPS+TEGO groups (Figure 4E, LPS+MP: 98.80 ± 9.28 and LPS+TEGO: 96.36 ± 9.35) compared to those in the LPS group (4.98 ± 0.11, *** *p* < 0.001). The gene expression levels of TGF-β also were significantly increased in the LPS+MP and LPS+TEGO groups (Figure 4F, LPS+MP: 5.49 ± 0.72 and LPS+TEGO: 2.90 ± 0.30) compared to those in the LPS group (0.36 ± 0.07, *** *p* < 0.001).

In other words, the gene expression levels of pro-inflammatory cytokines including IL-6, IL-1β, and TNF-α were most notably decreased in the LPS+TEGO group (Figure 4A–C), while anti-inflammatory cytokines including IL-4, IL-10, and TGF-β were significantly increased in the LPS+TEGO group (Figure 4D–F).

### 2.4. Effects of TEGO on the Phosphorylation of Mitogen-Activated Protein Kinase (MAPK) Signals

**Figure 5:**
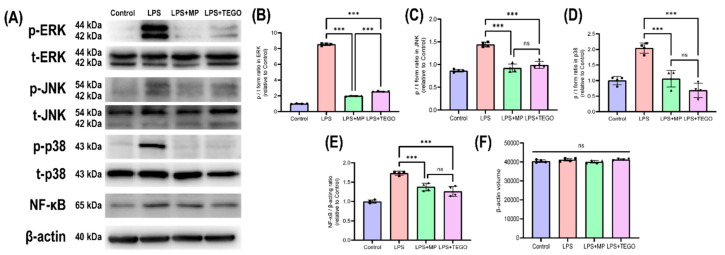
(**A**) Effect of TEGO on the phosphorylation values of extracellular regulatory kinase (ERK), c-Jun N-terminal kinase (JNK), p38, and nuclear factor kappa B (NF-κB) in BMMs stimulated by LPS. Quantitative analysis of the p/t forms of (**B**) ERK, (**C**) JNK, (**D**) p38, (**E**) NF-κB, and (**F**) β-actin. The results are expressed as the mean ± SD (n = 4 per group); not significant (ns); *** *p* < 0.001, a significant difference as compared to the control group and to each group.

The representative band images are presented in Figure 5A. The p/t values of extracellular signal-regulated kinase (ERK, Figure 5B, 8.54 ± 0.14) and p38 (Figure 5C, 1.05 ± 0.03) in the LPS group were significantly increased compared to those in the control (1.00 ± 0.03). On the other hand, the p/t form values of ERK were significantly decreased in the LPS+MP (Figure 5B, 1.99 ± 0.06, *** *p* < 0.001) and LPS+TEGO (2.54 ± 0.04, *** *p* < 0.001) groups compared to those in the LPS group (8.54 ± 0.14). The p/t values of c-Jun N-terminal kinase (JNK) were significantly decreased in the LPS+MP (Figure 5C, 0.92 ± 0.09, *** *p* < 0.001) and LPS+TEGO (1.00 ± 0.08, *** *p* < 0.001) groups compared to those in the LPS group (1.44 ± 0.05). Moreover, the p/t form values of p38 also were significantly decreased in the LPS+MP (Figure 5D, 1.06 ± 0.27, *** *p* < 0.001) and LPS+TEGO (0.69 ± 0.23, *** *p* < 0.001) groups when compared to those in the LPS group (2.05 ± 0.17). The NF-κB/β-actin values were significantly decreased in the LPS+MP (Figure 5F, 1.38 ± 0.10, *** *p* < 0.001) and LPS+TEGO (1.26 ± 0.13, *** *p* < 0.001) groups compared to those in the LPS group (1.74 ± 0.05). The β-actin, an internal control, was not different significantly between the groups (control: 40430.31 ± 723.97; LPS: 41139.31 ± 720.87; LPS+MP: 40048.80 ± 705.38; and LPS+TEGO: 41251.51 ± 386.01). In other words, the p/t form values of ERK and p38 were significantly decreased in the LPS+TEGO group (Figure 5B,C).

## 3. Discussion

This study determined the anti-inflammatory effects of TEGO in comparison to MP on LPS-stimulated BMMs. First, to avoid a cytotoxic concentration of TEGO, we utilized several concentrations (0, 0.0005, 0.001, 0.005, 0.01, 0.05, 1, 10, 100, and 1000 μg/mL) of TEGO to BMMs (Figure 2A). Cell viability in excess of 80% was considered as a non-toxic density level [22]. TEGO demonstrated no cytotoxicity up to 100 μg/mL. Based on this finding, we decided to use 0.5, 1, 10, and 100 μg/mL concentrations of TEGO in the subsequent experiments.

Macrophage colony-stimulating factor (M-CSF)-stimulated BMMs (M0-phenotype macrophages) can exhibit two distinct phenotypes: the M1 phenotypes (classically activated) and M2 phenotypes (alternatively activated). The M1-phenotype macrophages produce pro-inflammatory cytokines, whereas M2-phenotype macrophages produce anti-inflammatory cytokines [23]. In the various inflammatory environments, it is reported that most macrophages polarize toward the M1 phenotype, while the M2-phenotype macrophage is displayed in only a small number of cells [24]. Moreover, NO plays a role as a crucial inflammatory mediator to dilate the blood vessels, which induces infiltration of macrophages [25]. Pacher et al. reported that accumulated M1 macrophages exacerbate the pro-inflammatory response [26].

Here, we observed that NO production in LPS-stimulated BMMs was significantly inhibited by 100 μg/mL of TEGO (Figure 2B). Specifically, TEGO showed the ability to differentiate LPS-stimulated macrophages into M2-phenotype macrophages (Figure 3). These results indicated that TEGO can effectively suppress the production of NO in inflammatory cells and induce differentiation from M0-phenotype macrophages to M2-phenotype macrophages.

M1-phenotype macrophages are classically polarized by LPS and produce the typical pro-inflammatory cytokines, including TNF-α, IL-1β, and IL-6. On the other hand, M2-phenotype macrophages produce anti-inflammatory cytokines, including IL-4, IL-10, and TGF-β. We observed that TEGO downregulates the gene expression levels of pro-inflammatory cytokines and upregulates the gene expression levels of anti-inflammatory cytokines in LPS-stimulated BMMs. Sone et al. reported that the mRNA expression levels of pro-inflammatory cytokines, including TNF-α, IL-1β, and IL-6, were suppressed by treatment with TEGO in dinitrobenzene sulfonic acid-induced mouse models [27]. Collectively, our findings were similar to those of other recent studies, showing the potential anti-inflammatory effects of TEGO in the inflammatory response.

TEGO is a novel development as a P-CAB that inhibits gastric H^+^/K^+^-ATPase for the treatment of gastric-acid-related diseases, including gastroenteritis and GERD. Recently, it was reported that P-CABs show an anti-inflammatory effect by suppressing the MAPK signaling pathways [28]. Furthermore, Yeo et al. reported significant anti-inflammatory effects of PPIs by inactivating the Akt signaling, the NF-κB signaling pathway, and inflammatory cytokines in *Helicobacter pylori* infections that induce chronic gastric inflammation [29]. Collectively, our results and recent studies by others suggest that TEGO, already approved by the Korean FDA, can potentially be an NSAID for clinical use.

MAPK signaling is implicated in cellular processes including apoptosis, cell survival, stress response, and inflammation [30]. Among the MAPK signaling pathways, phosphorylation of ERK and p38 have an impact on the inflammatory response [31]. In this study, we conducted a further investigation of the signal pathways to investigate the anti-inflammatory effects of TEGO (Figure 5), showing that p/t volumes of ERK and p38 in the TEGO-treated BMMs were significantly decreased compared to those in the LPS-stimulated BMMs. Although the p/t volumes of ERK and p38 in the MP-treated group were the lowest among the groups, TEGO has the advantage of being suitable for long-term high-dose treatment [32]. Here, while we did not conduct in vivo studies, to the best of our knowledge, our study is the first to demonstrate that TEGO inhibits pro-inflammatory genes and induces anti-inflammatory genes when used on LPS-stimulated BMMs in vitro.

## 4. Materials and Methods

### 4.1. Preparation of TEGO and LPS

TEGO was purchased from Med Chem Express (Monmouth Junction, NJ, USA) and solubilized in dimethyl sulfoxide (DMSO, Thermo Fisher Scientific Inc., Waltham, MA, USA). For the in vitro method, the various concentrations of TEGO were diluted in Dulbecco’s modified Eagle’s medium (DMEM, Gibco, Thermo Fisher Scientific Inc., Waltham, MA, USA) including 10% fetal bovine serum (FBS, Gibco) and 1% penicillin-streptomycin (P/S, Gibco). LPS was obtained from Sigma Aldrich (Sigma, St. Louis, MO, USA). It was dissolved in distilled water and diluted with DMEM 0.1 μg/mL.

### 4.2. Isolation of BMMs and Differentiation into Macrophages

All rat experiments were performed according to the protocol approved by the Institutional Animal Care and Use Committee (IACUC) of CHA University (protocol code 220010, January 2022) and the Guide for the Care and Use of Laboratory Animals (National Institutes of Health, Bethesda, MD, USA). The isolation of the monocytes followed a previous method [8,33,34]. Briefly, the monocytes were extracted from the tibia/femurs of Sprague Dawley rats at four weeks. Afterwards, the monocytes were placed in a 100 × 20 mm petri dish (Corning Inc., Corning, NY, USA). The monocytes were cultured in DMEM including 10% FBS, 1% P/S, and 10 ng/mL of macrophage colony-stimulating factor (M-CSF, Peprotech, NJ, USA) in a 5% CO_2_ incubator at 37 °C. After 2 days of pre-culture, non-adherent cells in the supernatant were removed. Afterwards, adherent cells were considered macrophages. A detailed description of the isolation and differentiation of BMMs is provided in the Supporting Information.

### 4.3. Cell Toxicity Test

The cell viability kits used were obtained from EZ-Cytox (Daeil Lab Service, Seoul, Republic of Korea). BMMs (5 × 10^5^ cells/well, n = 4 per group) were seeded into a 96-well cell culture plate (Falcon Becton Dickinson, NJ, USA) and cultured with various concentrations of TEGO (0, 0.0005, 0.001, 0.005, 0.01, 0.05, 0.1, 0.5, 1, 10, 100, and 1000 μg/mL) for 24 h. After 24 h, BMMs were washed with Dulbecco’s Phosphate Buffered Saline (DPBS, Invitrogen, Thermo Fisher Scientific Inc., Waltham, MA, USA). Subsequently, the cells were incubated with free media containing cell counting kit-8 (CCK-8) for two hours. The intensity was determined with a microplate absorbance reader at a wavelength of 450 nm. Each observation of groups was normalized relative to the control group.

### 4.4. NO Production

The NO production measurements were performed as described previously [8,33,35]. Briefly, BMMs (1 × 10^6^ cells/well, n = 4 per group) were seeded into 48-well culture plates (Falcon) to prepare the seven experimental groups (control (only BMMs), 0.1 μg/mL of LPS, 1 μg/mL of MP, and 0.5, 1, 10, and 100 μg/mL of TEGO). After 24 h, NO products accumulated in the supernatant were detected using a Griess reagent system (Promega, Madison, WI, USA). Briefly, the supernatant and sulfanilamide were mixed in equal amounts. Each group was measured at a wavelength of 548 nm using a microplate absorbance reader (Bio-Rad, Hercules, CA, USA). The NO concentration in the supernatant fluid was measured with a standard curve generated with sodium nitrite.

### 4.5. ICC Staining

The ICC stainings were performed as described previously [36]. BMMs were seeded and cultured for one day in DMEM with MP (1 μg/mL) and TEGO (100 μg/mL) containing LPS (0.1 μg/mL). After one day, cells were fixed using 4% paraformaldehyde. Fixed cells were incubated with anti-CD86 and anti-CD206 antibodies at 4 °C overnight. Afterwards, Alexa 488 and Alexa 647 secondary antibodies were stained for two hours at room temperature. The nuclei were stained with 4′,6′-diamidino-2-phenylindole dihydrochloride (DAPI). The fluorescent intensity was detected using a Zeiss LSM 880 confocal microscope. A detailed description of the ICC staining methods is provided in the Supporting Information.

### 4.6. FACS Analysis

The FACS analysis was performed as described previously [36]. BMMs (1 × 10^6^ cells/well, n = 4 per group) were seeded into six-well culture plates (Falcon) and treated with each concentration of MP (1 μg/mL) and TEGO (100 μg/mL) containing LPS (0.1 μg/mL). After 24 h, cells were fixed with 4% paraformaldehyde and incubated with primary antibodies, including anti-CD68 (pan-macrophage marker), anti-CD86 (M1-phenotype marker), and anti-CD206 (M2-phenotype marker) antibodies for one hour. After washing, BMMs were stained with Alexa 488 and Alexa 647. The marker expression was analyzed on a CytoFLEX Flow Cytometer (CytoFLEX V5-B5-R3, Beckman Coulter, Brea, CA, USA), and data were analyzed using the CytExpert software 2.4 (Beckman Coulter).

### 4.7. qRT-PCR

BMMs (1 × 10^6^ cells/well, n = 4 per group) were seeded into six-well culture plates (Falcon) to perform the qRT-PCR analyses. Cells were treated with each concentration of MP (1 μg/mL) and TEGO (100 μg/mL) containing LPS (0.1 μg/mL). After 24 h, total RNA from seeded cells were extracted using Trizol reagent (Invitrogen) according to the manufacturer’s instructions. The qRT-PCR analyses were performed as described previously [8,33,34]. The primers were obtained from Bioneer (Daejeon, Republic of Korea). The relative gene expression values of IL-6, IL-1β, TNF-α, IL-4, IL-10, and TGF-β were normalized to those of glyceraldehyde 3-phosphate dehydrogenase (GAPDH) by using the 2-ΔΔCT calculation [37]. A detailed description of the qRT-PCR method, including the nucleotide sequences of primers, is provided in the Supporting Information.

### 4.8. Western Blotting

BMMs (1 × 10^6^ cells/well, n = 4 per group) were seeded into six-well culture plates (Falcon) and treated with each concentration of MP (1 μg/mL) and TEGO (100 μg/mL) containing LPS (0.1 μg/mL) for 24 h. After 24 h, cells were lysed by adding radio immune precipitation assay lysis buffer (RIPA lysis buffer, Sigma) with protease (Roche Applied Science, Indianapolis, IN, USA) and phosphatase inhibitor cocktail (Sigma) for 30 min at 4 °C. The p38 progressed 30 min after drug treatment. Subsequently, the protein was extracted by centrifugation at 13,000 rpm for 10 min. The concentration of the extracted protein was measured using a microplate absorbance reader (Bio-Rad) at a wavelength of 595 nm. The Western blot assay was performed as described previously [8,33,34,38]. The ratios of the phosphorylated form (p) over the total form (t) of ERK (p-ERK, 1: 1000; t-ERK, 1: 1000, Cell Signaling Technology, Danvers, MA, USA), p38 (p-p38, 1: 1000; t-p38, 1: 1000, Cell Signaling Technology), JNK (p-JNK, 1:1000; t-JNK, 1:1000, Cell Signaling Technology), and NF-κB (1:1000, Cell Signaling Technology) were quantified. Values of t forms were used as an internal control to quantify values of p forms. As an internal control, beta actin (β-actin, 1:5000, Abcam, Cambridge, UK) was also probed at the membrane. The visualized signal bands were detected using a horseradish peroxidase procedure with a ChemiDoc XRS System (Bio-Rad). The intensities of the bands for p and t forms were quantified using ImageJ at the National Institute of Health (NIH, Bethesda, MD, USA). A detailed description of the Western blot, including the antibody specifics, is given in the Supporting Information.

### 4.9. Statistical Analyses

Comparisons among each group were followed by a one-way analysis of variance (ANOVA), and Tukey’s tests were used as a post hoc analysis method. For *p*-values, * *p* < 0.05, ** *p* < 0.01, and *** *p* < 0.001 were considered statistically significant.

## 5. Conclusions

TEGO shows anti-inflammatory effects on LPS-stimulated BMMs. Although the anti-inflammatory effects of TEGO may impair the ability to defend against infection by suppressing the function of innate immunity, the results suggest that TEGO can be a useful non-steroidal anti-inflammatory drug.

## Data Availability

The data used to support the findings of this study are included within the article.

## Supplementary Materials

The following supporting information can be downloaded at: https://www.mdpi.com/article/10.3390/ijms241914589/s1.
