# Supplementary material for: Anti-Inflammatory Effects of Tegoprazan in Lipopolysaccharide-Stimulated Bone-Marrow-Derived Macrophages
Source: Int J Mol Sci. 2023 Sep 26;24(19):14589. doi: 10.3390/ijms241914589 (PMC10572893; doi:10.3390/ijms241914589)
Supplement: Supplementary file 1 [file ijms-24-14589-s001.zip › ijms-2621872-supplementary.pdf]

Supporting Information

Anti-Inflammatory Effects of Tegoprazan in Lipopolysaccharide-Stimulated Bone-Marrow-Derived Macrophages

Gong-Ho Han [1,2], Seong-Jun Kim [1,2], Wan-Kyu Ko [1,2], Je-Beom Hong [3], Seung-Hun Sheen [1], Min-Jai Cho [4,*] and Seil Sohn [1,*]

[1] Department of Neurosurgery, CHA Bundang Medical Center, CHA University, 59 Yatap-ro, Bundang-gu, Seongnam-si 13496, Gyeonggi-do, Republic of Korea; hgh429@chauniv.ac.kr (G.-H.H.); ksj987456@chauniv.ac.kr (S.-J.K.); wankyu@chauniv.ac.kr (W.-K.K.); nssheen@cha.ac.kr (S.-H.S.)

[2] Department of Life Science, CHA University, Boondagger, Seongnam-si 13493, Gyeonggi-do, Republic of Korea

[3] Department of Neurosurgery, Kangbuk Samsung Hospital, Sungkyunkwan University School of Medicine, Seoul 16419, Republic of Korea; jebeomhong@gmail.com

[4] Department of Neurosurgery, Chungbuk National University College of Medicine, Chungbuk National University Hospital, Seowon-gu, Cheongju-si 28644, Chungcheong-do, Republic of Korea

* Correspondence: c303004@cbnuh.or.kr (M.-J.C.); sisohn@cha.ac.kr (S.S.); Tel.: +82-31-881-7966 (S.S.)

1. Materials and Methods

1.1. Isolation of bone-marrow derived monocytes and differentiation into BMMs

Bones were cut in half and placed in a 1.5 ml reaction tube. Bone marrow was collected by centrifugation (5 min, 5000 × g). Cells were separated with a 40 μm cell strainer, and erythrocytes were lysed via hypotonic shock in sterile distilled water. To analyze adhesion and proliferation, we used an established protocol to enrich BMMs from whole bone marrow by their ability to attach to untreated plastic. Separation via adhesion to plastic provides easy and reliable access to the macrophage proportion of bone marrow cells. The obtained population is further considered to be BMMs. After centrifugation (5 min, 245 × g), the remaining bone marrow cells were suspended in 20 ml of macrophage medium. After 2 days of preculture, plates were washed with Dulbecco’s phosphate-buffered saline (DPBS, Invitrogen). The attached cells were detached by using 0.02% ethylene diamine tetraacetic acid (EDTA) in DPBS. Subsequently, 5 min incubation period on ice was followed by 1 min at −20 °C. After centrifugation and resuspension in DMEM, the cell number was determined with a hemocytometer.

1.2. ICC staining

The BMMs were seeded on confocal dishes (n=4) at a density level of 1×10^5 cells per dish and cultured with DMEM. After one day, the DMEM medium was replaced with LPS, LPS+MP, or LPS+TEGO for one day. Subsequently, the culture medium was removed, and cells were fixed with 4% paraformaldehyde for ten minutes. They were then washed with

DPBS three times and immersed in 0.2% triton X-100 for ten minutes. After washing three times with DPBS, the cells were blocked with a blocking solution (Invitrogen). The cells were then treated with anti-CD86 antibody and anti-CD206 antibody at 4 °C overnight. Afterwards, Alexa 488-conjugated and Alexa 647-conjugated secondary antibody was stained for two hours at room temperature. After washing the remaining secondary antibodies, the nuclei were stained with DAPI. For each confocal dish, the regions of interest (ROI) were randomly designated at 100X magnification. CD86 or CD206-stained area within the ROI image was quantified using the ImageJ software (NIH). The cells were detected using a confocal laser scanning microscope (LSM 880, Carl Zeiss).

1.3. qRT-PCR

Complementary DNA (cDNA) was synthesized from 1 μg of total RNA using a cDNA using synthesis kit (TAKARA, Shiga, Japan). The qRT-PCR step was performed using an ABI Step One Real-time PCR System (Applied Biosystems, Warrington, UK) and a reaction mixture that consisted of SYBR Green 2 × PCR Master Mix, a cDNA template, and forward/reverse primers. The primers of the measured mRNA genes were as follows: TNF-α; 5′ – AGC AAA CCA CCA AGT GGA GGA − 3′ (sense) and 5′ – GCT GGC ACC ACT AGT TGG TTG T - 3′ (antisense), IL-1β; 5′– CCC TGC AGC TGG AGA GTG TGG - 3′ (sense) and 5′ – TGT GCT CTG CTT CAG AGG TGC T - 3′ (antisense), IL-6; 5′ – TTG TTG CTG TGG AGA AGC TGT - 3′ (sense) and 5′ – AAC GTC ACA CAC CAG CAG GTT- 3′ (antisense), GAPDH; 5′ – ATG ATT CTA CCC ACG GCA AG - 3′ (sense) and 5′ CTG GAA GAT GGT GAT GGG TT - 3′ (antisense). The PCR

protocol consisted of 40 cycles of denaturation at 95 °C for 15 sec, followed by 60 °C for 30 s.

1.4. Western blot

At 24 h, the cells were gently scraped with a cell scraper (SPL, Seoul, Korea) and collected in e-tubes. In this case, 40 μg of resolved equal amounts of proteins were congregated to 10% of sodium dodecyl sulfate-polyacrylamide gel electrophoresis and transferred to nitrocellulose transfer membranes (Protran, Whatman, Germany). The membranes were incubated with 5% of skim milk for 1 h to block the nonspecific binding and probed with the primary antibodies of phosphorylated forms of the extracellular signal-regulated kinase (p-ERK), c-Jun N-terminal kinase (p-JNK), p-p38, and nuclear factor kappa B (NF-κB). Subsequently, equal membranes were stripped and reprobed with the total forms of ERK (t-ERK), JNK (t-JNK), and t-p38. All primary markers were followed by incubation with secondary antibodies (1:5000, Santa Cruz Biotechnology, Dallas, TX).
